# A meaningful daily life in nursing homes - a place of shelter and a space of freedom: a participatory appreciative action reflection study

**DOI:** 10.1186/1472-6955-13-19

**Published:** 2014-07-10

**Authors:** Inger James, Karin Blomberg, Annica Kihlgren

**Affiliations:** 1 School of Health and Medical Sciences, Örebro University, Örebro, Sweden

**Keywords:** Action research, Meaningful daily life, Nursing homes, Space, Freedom, Place, Security, Reciprocal relationship

## Abstract

**Background:**

Shortcomings in elderly care have been reported in many parts of the world, including Sweden. However, national guidelines for elderly care have been introduced in Sweden, which contain core values and local guarantees of dignity. These highlight the need for dignity and well-being, and organising the older person’s daily life so that they perceive it as meaningful. Therefore, the aim of the present study was to describe older persons’ experience and knowledge about obstacles, opportunities and solutions to developing a meaningful daily life for those living in nursing homes.

**Methods:**

This study combined the Participatory Appreciative Action Reflection (PAAR) and hermeneutic approaches. Twenty-five older persons participated and persons with dementia or cognitive impairments were included. Repeated interviews were carried out as reflective conversations, leaving 50 interviews in total, wherein the older persons provided their analyses and reflections on a meaningful daily life. Finally, an analysis of the data was completed based on a life-world hermeneutic approach.

**Results:**

We identified five tentative interpretations that describe obstacles, opportunities and solutions for a meaningful daily life. Themes 2 and 4 outline obstacles for a meaningful daily life, and Themes 1, 3 and 5 describe opportunities and solutions for a meaningful daily life: (1) Having space to be yourself; (2) No space to be yourself; (3) Belonging and security; (4) A feeling of insecurity; and (5) Longing for something to happen. In the main interpretation, we found that the five tentative interpretations are related to Tuan’s concepts of space and place, where place can be described as security and stableness, and space as freedom and openness.

**Conclusions:**

The reciprocal relationship is a solution for a meaningful daily life and occurs in the interaction between staff and older persons in nursing homes. It is the balance of power, and constitutes a place of shelter and a space of freedom for a meaningful daily life. The older person must have balance between shelter and freedom to have a meaningful daily life.

## Background

The numbers of older persons are increasing worldwide, and they can have, for example, chronic diseases, and loss of functions and social relationships, which increase their vulnerability and distress [1-3]. This can result in an extensive need for elderly care [4] such as long-term care in a nursing home [1,4].

The need for moving to a nursing home for support can be experienced as dependence [5-8], and along with the loss of physical function can lead to loss of autonomy [9], affecting dignity [6,7,9] and reducing freedom [10]. Bergland och Kirkevold [11] found that relationships with other older persons at nursing homes were an opportunity to have thriving experiences. Older persons experienced good care when staff took their requests and needs seriously, and invited the older person into a relationship [12]. Encounters with staff could reduce the amount of time spent alone and make daily life meaningful for older persons [13]. Staff can therefore be seen as a resource for meaning [14]. Haugan revealed the importance of the interaction between staff and older persons for meaning in life, hope and self-transcendence [15,16]. Perceived meaning in life for older persons in nursing homes is also significantly associated with the quality of life dimensions: physical, social emotional, functional well-being – and since meaning is an aspect of spiritual wellbeing it is closely related to spiritual well-being as well [17]. However, it can be difficult for older persons to find a meaningful daily life [18] in a nursing home due to shortcomings in elderly care, which have been reported in many parts of the world [1].

Hasson and Arnetz [19] reveal that older persons only receive good care in nursing homes when it came to their basic needs, and Hellström and Sarvimäki [8] and Harnett [20] found that the environment does not enhance individual autonomy. Harnett also found that staff used routines as power to control older persons and did not listen to their opinions [20], which can result in impersonal care [21]. Older persons’ daily life could be described as a life without a future, running the risk of deteriorating, falling into increased dependence*,* and getting closer to death [22], which can lead to hopelessness, helplessness and depression [23].

One obstacle to meet older persons’ extensive needs and provide good care can be the number of staff (often limited), which leads to older persons feeling vulnerable [24]. Staff can also be stressed and have difficulty keeping up with the work [8,24]. As a result, they may transfer their own values to the older persons, for example, by determining what is best for a person without regard for their opinions. Therefore, older persons’ opportunities to influence the care they receive become limited [25]. In Sweden, the shortcomings in elderly care have been noted and national guidelines have been introduced, which contain core values and local guarantees of dignity, and highlight the needs for dignity, well-being, and organising older persons’ daily life so that they perceive it as meaningful. The Swedish government has given each municipality the responsibility to formulate its own core values and local guarantees of dignity [25]. Based on this an interdisciplinary, collaborative action research project between Örebro municipality and Örebro University was designed and ran from September 2010 to January 2013.

The project aimed to seek obstacles, opportunities and solutions for developing a meaningful daily life from older persons’ perspective by working with them, their relatives, staff, and managers (James I, Blomberg K, Liljekvist E, Kihlgren A: Working together for a meaningful daily life for older persons: a participatory and appreciative action and reflection project—the lesson we learned. submitted). Another aim was to use the data to formulate core values and local guarantees. In this study, we focused on what we could learn from the older persons’ experiences and knowledge. Therefore, the aim of the present study was to describe older persons’ experience and knowledge about obstacles, opportunities and solutions to developing a meaningful daily life while living in nursing homes.

## Methods

This study combined the Participatory Appreciative Action Reflection (PAAR) [26] and hermeneutic approaches [27]. The PAAR approach, which is based on action research, was used when collecting data [26]. We used action research, as the methodological approach is to empower people to influence their own daily lives. This research has its roots in participation in thought and action with the participants [28]. According to Ghaye, what distinguishes PAAR from other action research is that the researcher has an appreciative perspective, which focuses on what is working well in practice. Ghaye states that if only problems are diagnosed within an organisation it takes energy from the system. However, the objective is not to overlook problems and deficiencies in the practice, but rather use a creative critical approach and pursue solutions with the participants in the research project [26]. It was not possible to invite all older persons because the number of participants would have become too big. Therefore a strategic selection of older persons ≥65 years of age living in six nursing homes was used to ensure variation [29]. Two of the nursing homes received favourable ratings, two received average ratings, and two received the lowest ratings in a recent user survey about quality of care [30]. Five nursing homes agreed to participate and one declined due to a heavy workload. The older persons received oral and written information from the manager at the nursing home, and the first five older persons from each unit who agreed to participate were invited. In nursing homes in the Örebro municipality the majority of older persons have cognitive impairments and 60% are diagnosed with dementia. However, these conditions could be in different stages. Based on this we included older persons who were able to express themselves verbally and understand the meaning of an interview, and did not exclude persons with dementia or cognitive impairments. Only after the older persons received information and signed our written consent did the researcher make contact. Throughout this research project, we ensured confidentiality for the participants. Altogether, 25 older persons participated, 15 women and 10 men, and their mean age was 92 (range 83–100 years).

### Ethical considerations

Including persons with cognitive impairment in a study can be regarded as unethical. However, since the project intended to develop elderly care, it may be regarded as unethical to exclude these persons. Szala-Meneok [31] argues that research with older persons may require special consideration, but people should not be excluded because of declining health status. A weak health consisting of cognitive impairments or dementia does not always mean that the persons could not express their experiences and knowledge. The older persons could withdraw their personal consent at any time during the study. It was, therefore, important to ask if they wanted to end the participation in the project and be sensitive and observe the individual’s body language. If in any way they expressed or signalled unease or discomfort in the interviews, we would have cancelled them; however, no interviews were cancelled. Ethical approval was granted by the Uppsala Regional Ethical Review Board (registration number 2011/009).

### Data collection

In this study, the participatory research was based on co-creating knowledge, where the focus was on the older persons’ experiences and knowledge about a meaningful daily life. Repeated interviews were carried out as flexible, reflective conversations (James I, Blomberg K, Liljekvist E, Kihlgren A: Working together for a meaningful daily life for older persons: a participatory and appreciative action and reflection project—the lesson we learned. submitted) [26]. It was important that the time between the interviews was not too long due to the risk that the participants would forget the prior conversation. Two research assistants and one researcher (IJ) carried out the interviews, and each older person dealt with the same researcher in the repeated interviews. The interviews were audio recorded. Since older persons in nursing homes can be expected to have multiple symptoms such as fatigue, pain, depression, and anxiety [4], the reflective conversations were conducted when the older persons had strength to participate in their home within the nursing home. The researcher (IJ), who according to the larger project also followed staff in their work, discovered that conversations with older persons with, for example, cognitive impairments, arise naturally during work; therefore, some interviews were conducted in relation to the observations. In order to co-create a deeper understanding of the older persons’ experiences and knowledge, it was important to strive to build a trustful relationship, and the researchers, in line with action research, aimed to learn from the older persons. Reflective open questions were asked about what daily life looked like for the older person and how a meaningful daily life could be developed. Positive issues for reflection were raised with regard to what opportunities might exist for a meaningful daily life. The questions included: what obstacles may be found in daily life? Do you have any suggestions as to how these obstacles can be avoided or resolved [26]? The interviews were transcribed verbatim by a secretary, documented chronologically, and compiled on an ongoing basis. For the next step, the recorded compilations were taken back to the older persons for the second interview. Some read the compilations; however, in most cases the researcher told the person about the content of what had been said. The participants provided their analyses and reflections on the content were made, as were changes when necessary. These conversations were also recorded, which led to a total of 50 interviews. Each interview took between 30 minutes to one and a half hours.

### Data analysis

Since action research does not have a specific analysis method and we wanted to obtain a more in-depth understanding of the older persons’ experiences and knowledge, we chose to use a life-world hermeneutic approach in the final analysis [27].

When the data collection was finished, the first author (IJ) read all of the transcribed interviews several times. An inner dialogue with the text was created to get an overall and preliminary understanding, ultimately a sense of the whole. The analysis continued by alternating between reading and understanding each part of the text in relation to the whole, at the same time the whole was understood in relation to the parts. Similarities and differences were looked for in and between the older persons’ descriptions about obstacles and opportunities for developing a meaningful daily life, and different assumptions and assertions were compared. Similar descriptions were grouped in five tentative interpretations (themes). Furthermore, each theme was examined for how it could be interpreted and understood. According to Dahlberg et al. [27], the meanings of the themes could also be illustrated by sub-themes. To strengthen the credibility all authors (IJ, KB, AK) searched for alternative interpretations using counterarguments in relation to the themes. When disagreement arose we discussed until we reached consensus. All of the tentative themes were found to be valid compared with each other.

Finally, a main interpretation was developed to summarise and explain the five tentative interpretations (themes) — the focus was to see something in a new way in order to deepen the understanding of it [27]. Several possible theoretical interpretations were critically proposed, and the interpretation that we found could best explain the data was Tuan’s concept of space and place, where place can be described as security and stableness and space as freedom and openness [32]. Throughout the whole interpretation, openness has been used in an effort to restrain preconceptions at the same time as we endeavoured to understand what lay beneath the surface to find the underlying meanings and make the “invisible” visible [27]. If interpretations were not judged to be credible, they were removed and others were tried [27].

## Results

The findings are presented in five tentative themes (interpretations) with sub-themes describing the older persons’ experiences and knowledge about obstacles, opportunities and solutions to develop a meaningful daily life. Themes 2 and 4 describe obstacles for a meaningful daily life, and Themes 1, 3 and 5 describe opportunities and solutions for a meaningful daily life. We conclude with a main interpretation in the discussion section since the main interpretation should explain the tentative themes. We used quotations to represent many older persons’ experiences and knowledge and establish the tentative themes. Words that have been omitted are indicated by (…) and square brackets [ ] are used for comments by the authors (see Table 1).

**Table 1:** The data analysis

**Sub-themes**	**Sub-themes**	**Sub-themes**	**Sub-themes**	**Tentative interpretation**	**Main interpretation**
***To manage on your own***	***To have it as usual***	***To long for appreciation***		***Theme 1. Having space to be yourself***	***In command of space***
To still be able to manage on their own.	To have everyday life run as usual.	To be appreciated and receive compliments from staff.		Having the sense that they are capable of things. A solution was to get space from staff to be themselves.	Being free and having command of the self.
***To not be able to do what you want***	***To adapt to others***	***To be lonely***	***To be questioned***	***Theme 2. No space to be yourself***	***Curtailed space***
Different diseases had affected them and their mobility was limited.	The older person had to wait for their turn.	To feel lonely, missing others and activities.	To have trouble with being questioned and reminded by staff.	Daily life was curtailed and they had lost themselves*.* An obstacle became that they did not have space to be themselves.	Others possibly curtailing our space.
***To sense reciprocity***	***To feel connected***	***To belong to the family***		***Theme 3. Belonging and security***	***To be at home***
Affection and reciprocity between staff and the older persons.	To feel connected with others and family. The mealtimes give a sense of belonging.	There was a sense of belonging among the persons who said they belonged to a family.		Being provided with a reciprocal relationship with staff and a sense of belonging with staff and others. A solution was to sense belonging, security and a feeling of home.	A place where one can feel attachment and find a shelter.
***No interaction***	***To be left alone***			***Theme 4. A feeling of insecurity***	***To be lost***
The older persons had no interaction with others. Staff had no time.	Some of the older persons had no or few relatives/friends who came to visit.			Losing others from no interaction and losing themselves. An obstacle was the feeling of insecurity.	A place where you experience loneliness and difficultly navigating.
***To have something to do***	***To learn new things***	***To go outside***	***To remember***	***Theme 5. Longing for something to happen***	***Freedom***
Something that interrupts daily life is a distraction and is rewarding.	To learn what had happened in society and in the world.	It did not have to be a major activity, even just sitting.	To be alone and remember what had happened earlier in life.	Occurrence interrupts the routines. A solution was having something happen.	Freedom implies an openness and participation with the outside world.

Development of sub-themes, tentative interpretation (themes) and main interpretation.

Themes 2 and 4 describe obstacles for a meaningful daily life, and Themes 1, 3 and 5 describe opportunities and solutions for a meaningful daily life of older persons.

### Theme 1: Having space to be yourself

#### To manage on your own

Several of the older persons said that a meaningful daily life was to still be able to manage on your own, take care of oneself, and not need too much help. They experienced this as meaningful: getting up in the morning and taking care of their own personal hygiene, dressing and going to breakfast, which was served in the dayroom: “*Meaningful? Yes, it’s that I can take care of myself – of course.*”

#### To have it as usual

It was also an opportunity for meaningfulness to have everyday life run as usual: “*It feels good with what you are used too.*” Their habits and routines were important to them. For example, it could be important to continue training to maintain or regain lost functions. Every mundane routine they performed was also seen as an exercise in itself. It was also especially important to keep traditions and celebrate the great feasts.

#### To long for appreciation

The older persons saw appreciation as an opportunity to receive compliments from staff and feel that they meant something. They said that they needed to be told that they had a nice apartment, had done something good or said something that staff would benefit from: “*It feels good to be appreciated by the staff, when they see that, I can still do something.*” It was also important that the compliments were genuine.

### Tentative interpretation

The older persons longed to be mobile and manage on their own as much as possible. To maintain daily routines and habits was an opportunity for a meaningful daily life. To know that everyday life belonged to themselves and that they still could decide their own day. Having the sense that they were capable of things, had value, and meant something to themselves and others was valuable. That staff appreciated them so they felt they were still someone to count on had meaning. An opportunity and a solution for a meaningful daily life were interpreted as having space to be yourself.

### Theme 2: No space to be yourself

#### To not be able to do what you want

One of the biggest obstacles for a meaningful daily life was that the older persons were not able to do what they wanted. Some revealed that it was difficult for them to find out what could give them a sense of meaning in daily life because they could not do anything. Different diseases had affected them, their mobility was limited and they needed lot of help:

“No, I do not know if I can say that it is good when you cannot do anything by yourself – no dressing or going to the toilet.”

They might even long to die when their body, in everyday life, was filled with pain:

“But there is evil everywhere in the body, the skeleton, and the belly. I have huge problems … It is quite unnecessary to have to live like this.”

The older persons also revealed that they could have difficulty seeing and hearing, which meant they could not talk to others, read or do handwork or other things. As a result, they could lose what had earlier been a major interest.

#### To adapt to others

The older persons had to adapt to staff and others in the nursing homes, which became an obstacle. Staff was stressed and had time constraints:

*“So when you want to say something [whistling sound] – then they are gone. Maybe* [I] *start to say two words and then they do not listen.”*

Staff had told the older persons that they had a lot to do, that they helped many others with getting up in the morning and provided food and medicines. This meant that the older persons had to wait for their turn, which could be difficult especially when the need was urgent:

“*But today it was a long wait, and I really had to go badly. It was not only pee … and then I have this stomach so I have to go to the toilet at once. And I alarmed and alarmed, and no one came.”*

They experienced this as not having freedom and being forced to comply with staff routines:

Older Person: “*Yes, I want to be free.*”

Interviewer: “*Can you feel free here?*”

Older Person: “*Not in any matter at all.*” They had to take turns with the other older persons in having their needs met.

Another obstacle to a meaningful daily life was not having the same staff on a regular basis, which occurs because agency staff was used or staff left and new people were hired. Several of the older persons said there was nothing “wrong” with the new staff, but they lacked the relationship and the affection they had felt from the staff that had left: “*There were four of them we really liked. And we had known them … we grieved for them … they knew* [how the older person want to have it in everyday life].”

#### To be lonely

A further obstacle was that the older persons felt lonely and the days were empty. Staff did not have time to sit down and talk or do something with them. The older persons said that the time passed without anything special happening. It was also pointless to ask for something special to happen for this could not be done: “*The staff does the best they can. More cannot be done, I believe. Life now, yes, it’s quit boring.*” There were no other older persons to do something with. The older persons also missed activities they had done before, activities that filled the day with joy, such as playing different types of games.

#### To be questioned

Some of the older persons found it was an obstacle and had trouble with being questioned by staff. Because they had lived a long life and had so many life experiences, it was particularly difficult to be questioned and reminded by staff when, from the staff’s perspective, they had forgotten something or said or done something wrong:

*“But it is clear that the only thing you hear* [as an older person] *is: Why did you say so? Why do you do so? It is that you will be reminded that you are doing something wrong or have done something wrong. Forgetting, this is very hard as one gets old … I am at least 90 years. There is quite a lot really.”*

### Tentative interpretation

The older persons’ different diseases had affected them, and their mobility and freedom was limited. They could not move or go anywhere so they could not do what they wanted. Staff was stressed and the older persons had to adapt to others and wait for their turn. Time passed and they were lonely, without any expectations and no one to rely on. Their daily life was curtailed and they had lost themselves*.* An obstacle for a meaningful daily life was interpreted as not having the space to be yourself.

### Theme 3: Belonging and security

#### To sense reciprocity

Older persons explained that the affection and reciprocity between staff and themselves was an opportunity for a meaningful daily life: “*Staff that you can be open with, where you give and take, it is lovely.*” Staff and the older person could joke with each other. What older persons particularly appreciated was not having to feel like they were a burden and disturbed the staff, or that they were worthless because they were old or in need of a great deal of help:

*“You’ve never seen or heard anyone being impatient in the least, no they say … just call* [the alarm]*, they say to me every day. And that’s what you do when there is something that you have* [to do]*.”*

The older persons experienced a friendly and caring staff: “*They are – yes you cannot imagine. So much love you get.*”

The older persons assigned their contact person, who was their own staff worker, a particular meaning and affection:

*“But she is great. Here she has me and another lady …* [the contact person] *is … happy and alert and organises and tinkers and decorates ….”*

The older persons especially appreciated if the staff could come into their room in the evening and sit down and talk for a while:

*“I have no one else here other then a couple of the girls* [staff]*; they are really sweet. We can talk with each other about everything. Coming in the evening when they come with medicine and then they sit down.”* The relationship with staff provided a sense of reciprocity.

#### To feel connected

Another opportunity for a meaningful daily life was to feel connected with others. Talking with other older persons in the nursing home provided a sense of communion. For some the conversations with others meant being human: *“Yes, it’s fun to be able to talk to people … it is not the amount that makes it,* [it means] *that I am a human.”*

Affinity was created during the time they had coffee together, listened to music, and joked with each other. It could also happen at a party:

*“Last Saturday at least we had a 95*^*th*^*birthday party. So then we had the dinner in the dining room. Yes, there was some fun. It was solemnly.”*

One opportunity was seen when chemistry occurred between persons: “*You must like each other.*” Several older persons reported that they had learned to thrive in the nursing home and it felt like home: “*Because then, I was more at home here. And it was the best that could happen, I think.*”

A mealtime with good tasting food was also seen as an opportunity for a meaningful daily life and gave a sense of being connected: “*The food is important, you know, when not much is not happening.*” Sitting down at a table, not having anything to worry about and to be served could create a sense of security. They also appreciated being able to get a snack in the afternoon:

“And you can take a little extra if you want in the afternoon. They always have something, if you say: Can I have that and that? Well then you get it.”

Some of the older persons said that staff could also bake cakes and they especially appreciated having them: “*So we are a little spoiled.*”

#### To belong to the family

To belong to a family was an opportunity for a meaningful daily life and the older persons longed for their family visits. This was the greatest joy and gave meaning in everyday life. The older persons wanted to offer coffee and hear news about something special that had happened to mutual friends or neighbours: “*It is first and foremost if they have heard any news. So then they tell. And then the kettle is on.*” Another way to stay connected and share events was by telephone: “*I call. If no one calls me, then I call.*”

### Tentative interpretation

The older persons felt cared for by staff, and there was a relationship of devotion and reciprocity between staff and the older persons. There was also a sense of communion with the other seniors that tied everyday life together. The sense of belonging, the reciprocal relationship and devotion to others, including family and other older persons in the nursing home, created a familiar security. An opportunity and a solution for a meaningful daily life were being interpreted as a sense of belonging and security of feeling at home.

### Theme 4: A feeling of insecurity

#### No interaction

An obstacle was when the older persons had no interaction with others. They revealed that there were few other older persons to feel connected to, talk to, or share common interests with. Some older persons did not hear or see or have conversations: “*No, you understand, they’re as crazy as all of us, like me. No, you have nothing to talk to them about.*” In some cases, older persons felt that certain older persons were so “dizzy” that they should be in a nursing home designed for people with dementia. It could also be that the older persons had no desire to talk to anyone. Days passed without many interactions. Staff also had difficulty finding time to talk to the older persons. Nothing was going to happen as it did in everyday life and there was no expectation, and some older persons longed for death: “*I wish I could have avoided this, but I do not want to go away from my children.*”

#### To be left alone

Another obstacle was being left alone in life. Some of the older persons had no or few relatives who came to visit, or no friends to talk to or call. Former friends might be dead or lost through dementia:

“We have called each other in the last years because we do not meet. As it was, she was gone when she could not talk on the phone anymore, but she is still alive — 101 years she is — but we are losing each other.”

### Tentative interpretation

The older persons had no one to talk to, and some did not hear or could not conduct a conversation for other reasons. Staff could also be so stressed that they did not have time to stop and talk, so there was no interpersonal interactions. An obstacle for a meaningful daily life was interpreted when the older person had a sense of losing themself, which created a feeling of insecurity.

### Theme 5: Longing for something to happen

#### To have something to do

It was an opportunity to have something to do that interrupts daily life. It could be things like taking a walk, going to the gymnasium, or playing any sort of game such as bingo. It was not certain that it was bingo itself that was rewarding, but that the people who led the activity were nice and in some cases handsome. Activities could lead to older persons having more energy: “*Then I think you get a little better like that, you get some speed in yourself …*” What could also be rewarding was reading a magazine or watching the Royals and discussing their dresses: “*There is Victoria* [the crown princess of Sweden]*. She is always pretty.*” Reading or listening to a book and watching TV also provided a distraction and could fill the day with meaning.

#### To learn new things

The older persons wanted to have the opportunity to learn new things, hear the latest news, what had happened in the neighbourhood, society, and even in the world. They wanted to know what was going on. It also emerged that they missed activities like a study circle where they could learn about different things. However, there were some obstacles to this because they felt that few other older persons were able to attend such activities because they were too old and sick:

“But there are so many old ones here, so that they cannot cope with so many activities, right. So then there will be nothing out of the ordinary.”

#### To go outside

It was an opportunity for meaningful daily life to go outside and be able to sit in the sun and drink coffee: “*Well now I’m waiting for us to go outside, and they say they have coffee service. In the summer you drink coffee.*” The older persons wanted to be outside when the family and children came. It did not have to be a major activity, but just sitting in the sun and leaning against a wall, for example. There were also older persons who longed for the traditional tours that take place in the nursing home. Those who did not want to go outside or go on excursions were those who felt that they could not cope with anything anymore. The older persons’ stories were also about the freedom they had experienced earlier in life when they could go on excursions on their own:

“Yes, I would love to go, for example, as I did before when I had my sister and brother-in-law. Then we go to the national park. And it is lovely. We were often there when the primroses were there and the ground was yellow.”

#### To remember

Some perceived it as an opportunity to be alone and remember what had happened earlier in life. In their mind they thought of the people they had lived with, met, and had as neighbours. Their experience was that time was moving faster.

Interviewer: *“This to keep on and think over your life, do you think that is important?”*

Older person: *“Yes I think so. And I think about all my old neighbours and all old acquaintances … up through the road where I lived and what they were called and … how it was.”*

It could also be meaningful to remember things that they had done (or that they imagined they were doing):

“Then I take the bus to my parents sometimes and so on. And then I have a boyfriend that I am together with and so and we have a nice time.”

### Tentative interpretation

The older persons longed to have something that interrupts their daily routines. Having a meaningful daily life meant to have something happen: to play games, go to bingo or do exercises. It did not always matter so much what it was, just that something happened. It was also seen as an opportunity for meaningfulness to receive news and learn of what was happening in the outside world and the local community. Being alone and remembering or imagining could provide meaning. The older persons also longed to go outdoors and sit in the sun and drink a cup of coffee. These occurrences interrupt the usual routines and patterns, which create a sense of freedom for a while. An opportunity and a solution for a meaningful daily life were interpreted as longing for something to happen and to be a part of it.

### Main interpretation

According to the reflective life-world research, the intention of the theoretical main interpretation is to expand on and draw conclusions with regard to the earlier tentative interpretations (themes) [27]. In developing the main interpretation, we found that the five tentative interpretations could be related to Tuan’s [32] concepts of space and place, because space and place are components of an environment such as a nursing home in this case. Furthermore, the focus is on the human experience, which could be linked to the older persons experience and knowledge.

The concepts space and place are intertwined, each needing the other to be able to explain and define the other. Place can be described as security and stableness, and space as freedom and openness, wherein space is more abstract than place. Tuan means that we are attached to a place and long for freedom, as people need both place and space. We live in a dialectical movement between shelter and venture, attachment and freedom [32]. The first two themes and the last one we interpreted are related to the concept of space. Theme 1: Having space to be yourself can be linked to the concept in command of space, which is explained as being free and the self having command. Theme 2: No space to be yourself can be linked to the concept of curtailed space, which can be understood as others possibly curtailing our space. Themes 3 and 4 are interpreted as related to the concept of place. Theme 3: Belonging and security can be linked to the concept to be at home, where home is a place one can feel attachment and find a shelter; and Theme 4: A feeling of insecurity can be linked to the concept of being lost, meaning a place where you experience loneliness and difficultly navigating because you do not know the paths. Finally, Theme 5: Longing for something to happen can also be linked to space and the concept of freedom, where freedom implies an openness and participation in the outside world [32]. The main interpretation with the five tentative interpretations of the results will be discussed, in order to see something new [27], through Tuan’s concepts [32].

## Discussion

### In command of space

One opportunity and a solution for a meaningful daily life for the older persons was to have space and be themselves so that everyday life belonged to them, in other words they were in command of space. They longed to be mobile and manage on their own. Tuan outlines that the upright human body is the starting point for us to be ready to act. There space completely opens up in front of us, behind us and to the right and left [32]. To still be able to have a usual life and manage the everyday by keeping routines may lead to the older persons being able to keep their lifestyle and self-identity [33], and in that way be in command of space and feel free [32]. At the same time as the older persons’ longing for freedom and remaining in command of space, their space was curtailed.

### Curtailed space

The main obstacle was the different diseases affecting the older persons. Their mobility was limited and they needed a great deal of help. Tuan [32] describes the opposite of the upright body as being the back that can be perceived as the dark and the past, being in the shadow. Being unable to move around and go where you want limits a person’s power of action and makes them unfree. The older person’s body can take a lot of space and by curtailing that space it can be seen as an obstacle to a meaningful daily life. The older persons might have felt as they were in limbo in their body [34], or “stopped within a track” [35]. This could result in difficulties to communicate with body language. Zaletel, Kovacev, Sustersic and Kragelj [36] found that older persons make greater use of facial expressions and head movements than gestures or body movements when they communicate. Since body language is so much of our communication, the question becomes how older persons can mediate themselves. It would be a challenge for staff to be responsive to the older person’s body language. Moreover, the staff’s body language is significant because they can curtail the older person’s space in their shared space. The older persons also felt themselves questioned by staff. If staff see themselves as the one who has knowledge and is in charge, their body language can become expansive and take more space; they can take command of space and curtail it for the older person. However, it can be difficult for staff to be responsive under stress [24]. Time constraints and stress resulted in the older persons adapting to the other older persons and waiting for their turn to have their needs met. In addition, they felt that staff doubted them [24]. The older persons had understood that their space was being curtailed. There is a point where spaciousness becomes crowding, and it is different for all of us [32]. Everyday life had a curtailed space and the older persons had to adapt to the nursing home’s routines. Adherences to routines are also shown in other studies [8,20,37]. Questions are raised about why older persons comply. According to Tuan [32], humans organise space based on their experiences of their body and of others to suit their biological and social relationship needs. The older persons can, in other words, try to please others [8] or adapt to current routines to maintain their dignity [7]. The individual may feel better with complying than with making demands [12]. There may be a need to obtain affirmation and a sense of belonging; without adaptation there is no belonging and no space. However, in this study adaptation is seen as an obstacle to a meaningful daily life. Not being able to decide about everyday life can lead to resignation and a sense of worthlessness [8].

### To be at home

Humans can crowd our space but also enlarge our world, heart and mind [32]. The older persons found a place in nursing homes, and they felt secure and at home due to staff’s actions. It was the relationship between staff and older persons that constituted attachment, security and a shelter. Tuan [32] refers to Tennessee Williams, who argued that a feeling of being home may in fact be another person. Relationships and reciprocity was a solution for a meaningful daily life. Good relationships with staff can create a sense of belonging and value [6], make life easier, more joyful and meaningful for the older persons [19,38], and satisfy basic psychological needs, which contribute to well-being [39]. It is in the relationship with staff that autonomy, self-determination and identity can be maintained [40].

That staff came to represent stableness and a place of security raises the question: why is it not the physical place of a nursing home itself that represents security? Tuan [32] argues that it takes a long time to get to know a place. Ordinary housing has furniture like a table, chairs and sink that are points on a complex path. Through habit the path acquires “a density of meaning and stability” as characterising the place. The sense of a place needs to be experienced day after day for several years until it settles in the body, which can be seen in body language. It could be that the relationship with “the home” was reflected in the staff’s body language who mediated a positive interest and engagement. A smile mediated happiness and well-being. When meeting others, one feels happier and more connected to a person who is smiling [32,41]. Through interaction with the older person staff can mediate a sense of home, which can create shelter and security [32] and a meaningful daily life. This increases physical and, mental, well-being and psycho- spiritual functioning among the older persons’ [14].

### To be lost

Longing to be in command of space, as the older persons expressed, can also mean a threat, resulting is being exposed and vulnerable [32]. The vulnerability is also described by the older persons in this study as being alone and isolated in their nursing home. Even if moving to and living in the nursing home is voluntary [42,43] and within the same city, it can still mean a big change, which can affect social identity and relationships [44]. To meet a stressed staff member who does not have time to talk [24] may increase alienation when the older persons may not have received any help to orient themselves to the new place. If they had no one to talk with to obtain clues about how they should behave, this could mean that the older persons feel as if they are nowhere. In Tuan’s perspective, this means that older persons do not recognise the place. There are no incorporated paths to walk on and they do not know how to navigate [32].

How can the older persons’ home and shelter remain a possibility and the obstacle of being lost be overcome? The older persons in this study provided solutions for this. They experienced speaking to staff about everything and received a lot of love. The relationship was fundamental and was characterised by reciprocity. This can be described as a form of partnership, and together they made everyday life work [45]. According to Ploeg et al., it is the relationship that staff creates with older persons and their families that can enhance the quality of care [46].

Tuan [32] argues that the intimacy and closeness that occurs in the nurturing relationship “in the home” does not mean the people need to know each other well. It glows in moments of true awareness and exchange. This reciprocal relationship is an opportunity for older persons and staff to open the window to the environment or outside world, and to a new venture and freedom.

### Freedom

A meaningful daily life meant satisfying the longing for something to do, something to happen and being free. It could mean that daily life would not be as organised or structured as in nursing homes. Tuan [32] explains that “open space has no trodden paths and signposts” (p. 54). The horizon is free and open. While nursing home staff offered many organised and structured activities, it seems that older persons were asking for activities of a more individual nature. Therefore, the range of activities must be wide enough to accommodate the needs of people with different backgrounds and interests [47]. When activities are based on a person’s interests, the activity becomes a freedom. The interaction between the nurse and the older person and activities in nursing homes can increase mental, physical and psycho spiritual well-being, and prevent despair and meaninglessness [14]. Furthermore, the interaction between the nurse and the older person significantly affects nursing home patients’ depression and anxiety [48].

Another way to feel free could be to go outside and sit or lean against a wall and let one’s thoughts wander freely. Furthermore, thinking of the past and remembering what has been or creating memories with the help of imagination can be a way to feel free. To remember could be to forget the present, which could mean freedom. Tuan considers that all people need to look back to acquire a sense of self and identity. The older person is more than what the thin present defines them as [32], as life history may be of significance [33]. The older persons may tell their life history freely with no beginning and no end, perhaps as an imaginary story, and if staff comes along “on the trip in a reciprocity relationship” a meaningful daily life can be created.

### Methodological consideration

In action research it is important that the participants participate as co-researchers [49]. A limitation in the present study is that the older person can be seen as co*-*researchers to only a certain extent in the collection of data. Thus, they were able to change, delete or advise shortcomings, or add to the material. However, the role of the researcher may be to draw conclusions from the research data [50], for example interpret what the older person has said in relation to Tuan’s concepts. However, the research was also associated with some difficulties, as some of the older persons had trouble keeping the “thread” through the conversation. To handle this the researchers waited for the older persons when they had difficulty expressing themselves verbally, followed them into other stories and then returned the conversation back to their experiences of a meaningful daily life. When the older persons became tired the researchers stopped the conversations and returned another day. The older persons appreciated to participate in the research. The interviewers were all nurses with long experiences in elderly care and communicating with older persons with dementia. The repeated interviews and going back several times to conduct reflections deepened the knowledge of a meaningful daily life of older persons living in nursing homes. This can be seen as a form of validation [51] and is a strength in the study. During the interpretation process we tried to maintain a bridled attitude [27], and be open to the “data”. To strengthen the trustworthiness, we critically tested other possible theoretical interpretations. However, from our perspective the main interpretation in this study is based on Tuan’s concepts of place and space, which could best explain the results [27].

## Conclusion

**Figure 1:**
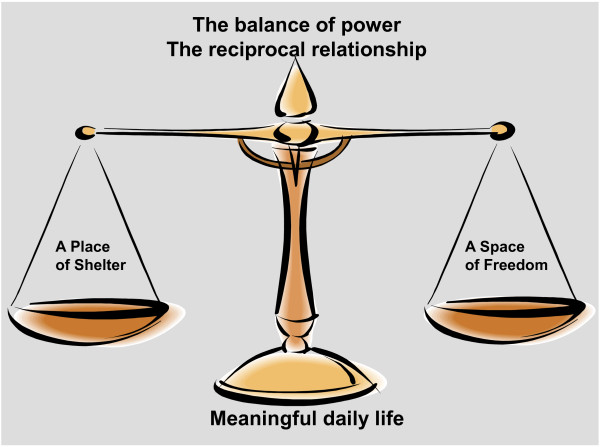
**The reciprocal relationship is the balance of power.** The reciprocal relationship is a solution for a meaningful daily life and occurs in the interaction between staff and older persons in nursing homes. It is the balance of power, and constitutes a place of shelter and a space of freedom for a meaningful daily life. The older person must have balance between shelter and freedom to sense a meaningful daily life.

From the older persons’ experiences and knowledge we learned that obstacles for a meaningful daily life included older persons being unable to do what they wanted, and having to adapt to staff and routines. The older persons explained that the staff did not have time to sit down and talk or do activities with them, and that they also had trouble with being questioned by staff. There was no interaction with others and they were left alone; they did not have space to be themselves. They were losing others because of a lack of interpersonal interaction. They were also losing themselves and felt insecure. According to Tuan [32], this can be interpreted as their space being curtailed and a feeling of being lost. However, from the older persons’ experiences and knowledge we also learned about opportunities for a meaningful daily life: opportunities to manage on your own, to live their usual lives, and to be appreciated. The older persons experience affection and a reciprocal relationship between themselves and staff, feel connected with others, and feel a belonging to family. Opportunities included having something to do, learning new things, going outside and remembering, all of which implies freedom. According to Tuan [32], this can be interpreted as being in command of space, feeling secure and finding a shelter, and having a sense of freedom, which were opportunities and solutions for a meaningful daily life. Based on the results we can conclude that it is the nursing home staff and the reciprocal relationship they create with older persons that is the difference between obstacles and opportunities for a meaningful daily life. The reciprocal relationship that occurs in the interaction between staff and the older persons balances power and constitutes a place of shelter and a space of freedom. Older persons must have a balance between shelter and freedom to sense a meaningful daily life (see Figure 1). We find support for our findings in Haugan’s studies that show the effect of the interaction between nursing home staff and older persons and meaning in life [15,16]. It prevents despair and meaninglessness [14], and has a significant affect on nursing home patients’ depression and anxiety [48]. To have the reciprocal relationship and their interactions become an opportunity and a solution, staff must understand their own values and balance their power in the relationship. Their own values guide their actions, which are reflected in their body language [52]. They should hold values that allow older persons to have command of space, feel at home and have freedom. The power to make decisions must be handed over to older persons in order to make an impact on their own daily lives. It is essential that staff have more training in reciprocal relationships to balance the power in their interactions, and create a place of shelter and a space of freedom for a meaningful daily life for older persons in nursing homes.

## Competing interests

The authors declare that they have no competing interests.

## Authors’ contributions

IJ, KB and AK designed the study and drafted the manuscript. IJ made the initial analysis of the interview transcripts. Each step of the analysis was then scrutinised and discussed by IJ, KB and AK. Furthermore, IJ, KB and AK made critical revisions to the manuscript, and all of the authors read and approved the final manuscript.

## Pre-publication history

The pre-publication history for this paper can be accessed here:

http://www.biomedcentral.com/1472-6955/13/19/prepub
